# Broad attention uncovers benefits of stimulus uniformity in visual crowding

**DOI:** 10.1038/s41598-021-03258-z

**Published:** 2021-12-14

**Authors:** Koen Rummens, Bilge Sayim

**Affiliations:** 1 Institute of Psychology, University of Bern, Bern, Switzerland; 2 UMR 9193 - SCALab - Sciences Cognitives et Sciences Affectives, Université de Lille, CNRS, 59000 Lille, France

**Keywords:** Human behaviour, Object vision

## Abstract

Crowding is the interference by surrounding objects (flankers) with target perception. Low target-flanker similarity usually yields weaker crowding than high similarity (‘similarity rule’) with less interference, e.g., by opposite- than same-contrast polarity flankers. The advantage of low target-flanker similarity has typically been shown with attentional selection of a single target object. Here, we investigated the validity of the similarity rule when broadening attention to multiple objects. In three experiments, we measured identification for crowded letters (Experiment 1), tumbling Ts (Experiment 2), and tilted lines (Experiment 3). Stimuli consisted of three items that were uniform or alternating in contrast polarity and were briefly presented at ten degrees eccentricity. Observers reported all items (full report) or only the left, central, or right item (single-item report). In Experiments 1 and 2, consistent with the similarity rule, single central item performance was superior with opposite- compared to same-contrast polarity flankers. With full report, the similarity rule was inverted: performance was better for uniform compared to alternating stimuli. In Experiment 3, contrast polarity did not affect performance. We demonstrated a reversal of the similarity rule under broadened attention, suggesting that stimulus uniformity benefits crowded object recognition when intentionally directing attention towards all stimulus elements. We propose that key properties of crowding have only limited validity as they may require a-priori differentiation of target and context.

## Introduction

In real-world settings, the amount of visual information is often overwhelming. Selective attention helps to prioritize information in the visual environment that is most relevant to current behavioral goals, while ignoring distracting information^1^. Observers’ deployment of attention can thus strongly alter the effect of irrelevant information on performance in many visual tasks. For instance, visual crowding, i.e. the interference of task-irrelevant close-by items (flankers) with object perception^2–8^ (for reviews see^9–12^), has been shown to depend on the spatial allocation of attention. When a pre-cue indicated the location of a crowded target, identification was enhanced relative to a no-cue condition^13–17^. These findings were attributed to observers’ deployment of spatial attention, with reduced crowding when attention was focused around a limited region. Benefits of focused attention were also revealed when unilateral compared to bilateral stimulus presentation yielded weaker crowding^18^. Furthermore, attention has been suggested to underlie one of the key characteristics of crowding, i.e., its inward-outward anisotropy, where a peripheral flanker interferes more strongly with target perception than a foveal flanker. Inward-outward anisotropy was observed when attention was focused around a fixed target location, but disappeared when attention was diffused over several possible target locations^19^. In contrast, other studies did not reveal similar modulations of crowding by spatial attention, revealing that neither precuing the target location^20,21^ nor varying attentional focus affected crowding^22^. Hence, effects of spatial attention on crowding are equivocal. In the current study, we aim to further clarify the link between attention and crowding.

Crowding is characterized by a number of often-replicated properties, which have obtained the status of ‘rules’^23–26^. One of the central rules of crowding is its dependence on target-flanker spacing. The critical spacing—the distance at which radially positioned flankers start to impair performance—has often been estimated to be about half the target’s eccentricity (‘Bouma’s law’^2^). Within Bouma’s range, flanker interference is usually more severe at smaller than at larger distances from the target^27^. Another key property of crowding is its dependence on the similarity between the target and flankers, with flankers that are more similar to the target typically yielding stronger crowding than less similar flankers (e.g.,^27,33,36,39^). Exemplary for the ‘similarity rule’ is the usually better identification of a crowded letter with flanking letters of opposite compared to same contrast polarity (i.e., the ‘polarity advantage’^28–32^). Similarly, crowding decreased when targets and flankers differed in binocular disparity^28,33,34^, color^28,34–36^, orientation^37–39^, or shape^20,28,40^. Furthermore, when target-flanker similarity varied on multiple dimensions (color, spatial frequency, and orientation), crowding weakened as the number of feature dimensions on which target and flankers differed increased^41^. Target-flanker similarity was also suggested to operate at a higher, categorical level: Crowding of a target letter was more severe when flanked by letters than by numbers^42^, even when featural differences were controlled for^43^ (but see^44^).

However, neither similarity nor spacing between the target and its immediate flankers reliably predict crowding strength. For example, when measuring offset discrimination for a black vernier flanked by ten lines on each side, performance was superior with uniform white compared to alternating white and black flankers^34^. Importantly, the innermost flankers (to the left and right of the black target) were white in both conditions, suggesting that performance depended on the global stimulus configuration (i.e., target and flanker arrays) rather than local context (i.e., target and innermost flankers). The results were attributed to target-flanker grouping: When the flankers on each side of the target grouped amongst each other but not with the target, the target “stood out” from the flankers, yielding superior performance compared to when the target grouped with the flankers and did not stand out. Importantly, a few studies quantified target-flanker grouping with additional measures, complementing the crowded identification or discrimination tasks. In particular, subjective measures of target conspicuity were shown to predict crowding strength: Targets that were rated to stand out more strongly from the flankers were also less crowded^45,46^. Similarly, objective measures of how much a target stood out from the flankers have been shown to predict performance in crowding tasks: Targets that ‘popped out’ in a visual search task were less crowded in a discrimination task with the same stimuli (and known target location)^13,47^. Finally, contextual modulation itself has even been proposed as a measure of grouping strength, suggesting good (bad) performance in a crowding task to be indicative of weak (strong) target-flanker grouping^40^. In general, the rule of target-flanker grouping typically predicts more severe crowding when target and flankers form a coherent perceptual group (‘strong grouping’) than when the target can be easily segmented from its flankers (‘weak grouping’)^34,35,45,48,49^ (see^9^ for a review).

Limitations to the generality of Bouma’s rule were suggested by, for instance, its dependence on the density of the display^25,50^. While Bouma’s rule applied in sparse displays, crowding in densely cluttered displays seemed to depend only on the target’s ‘nearest neighbors’, i.e., those flankers that were within a radius far smaller than Bouma’s range. Furthermore, items at larger than critical spacing have been shown to modulate crowding^48,51^, and the presence of more versus less flankers within Bouma’s range can also alleviate—instead of increase—crowding ^35,45,48^. More recently, clear exceptions to the rules of spacing, similarity, and grouping were demonstrated. Melnik and colleagues^52^ revealed weaker instead of the usual stronger crowding at smaller than at larger target-flanker spacing when the target and a flanker combined into a configuration with a salient emergent feature. In particular, the typical effect of target-flanker spacing did not hold when a central target chevron was flanked by four chevrons, one of which formed a diamond-like shape with the target or was of the same orientation as the target. Thus, at close target-flanker distance, the gain in task-relevant information provided by the emergent feature of the target-flanker combination (i.e., closure or translational symmetry) seems to have counteracted the usually stronger crowding with closer than more distant flankers. Furthermore, emergent features were suggested to override the similarity rule of crowding. In a recent study, tilt identification (left- or rightward from the vertical) of the central line within a line triplet with unidirectional flankers (\\ or //) was similar with opposite- compared to same-contrast polarity flankers^53^. The absence of the polarity advantage was further investigated with an odd-quadrant task, revealing easier discrimination between uniform compared to alternating line triplets with unidirectional flankers (e.g., \\\ and \/\). These findings suggested that emergent features benefitted performance for uniform triplets more than for alternating triplets, counteracting the typically strong crowding between same-contrast polarity lines and enabling a similar performance level as with opposite-contrast polarity flankers. Similarly, emergent features were attributed a key role in the inversion of the similarity rule when observers were more accurate at identifying a diamond shape among highly similar diamond flankers compared to flankers consisting of dissimilar Xs^23^. Taken together, the violations of basic crowding rules suggest that strong grouping can also benefit performance by enhancing the availability of task-relevant information, enabling similar or even better performance compared to weak target-flanker grouping.

The effect of target-flanker grouping—either deteriorating or improving performance—thus seems contingent on task-relevant information provided by flankers when combined with the target. Specifically, strong target-flanker grouping may hinder selective attention towards the target only, and promote unintentional processing of the flankers. When context is uninformative on target identity, such unintentional processing of flankers has been suggested to interfere with target recognition in peripheral crowding experiments as well as in foveal flanker tasks (e.g.^34,54–56^). However, when strong grouping instigated the processing of informative (but otherwise task-irrelevant) flankers or target-flanker combinations, performance in crowding tasks was equal or better than when grouping was weak^23,52,53^. Hence, in order for conventional crowding rules to hold, it may be a prerequisite that the involuntary processing of flankers—under strong target-flanker grouping—does not enhance the availability of task-relevant information. As attention is ideally directed towards the single target item but not the surrounding items, optimal performance in typical crowding tasks would require rather narrow attention. In contrast, many tasks in real-world settings require broader attention as targets often are not pre-defined and usually more than one item is task-relevant. Previous studies suggested that effects of target-flanker similarity may be different when attention needs to be broadened to multiple task-relevant items instead of focused on a single one only. Indeed, recognition of the central letter among all black trigram letters was better when observers had to report all three letters (full report) instead of only a single letter (single-item report)^64^ (but see ^65^). The validity of the similarity rule was also suggested to depend on attentional allocation, as central trigram letter recognition was superior with opposite versus same-contrast polarity flankers in single-item report but only minimally better in full report^30^. Similarly, word recognition and reading—tasks that also involve broadened attention as identification of multiple letters is required—was not enhanced when word parts alternated in contrast polarity compared to when word parts all had the same contrast polarity^30,32^. Hence, the validity of basic crowding rules may therefore be limited to the specific case in which a single visual target in peripheral clutter is pre-defined, and any benefit of broadening attention towards multiple items is absent.

In the current study, we investigated the validity of the similarity rule in the absence of any a-priori segmentation of what constitutes target and context. Specifically, we examined how broadening attention to several instead of a single crowded target modulated the effect of target-flanker similarity. In three experiments, we compared the effect of a typical target-flanker similarity manipulation, i.e., opposite versus same contrast polarity, when attentional selection of either a single or multiple crowded items was needed. To this end, in Experiment 1, observers were instructed to report either all three letters (i.e., full report) or a single letter (i.e., single-item report) of a letter trigram, demanding surrounding items to be processed or not. For both report types, trigram letters were either uniform (all black or white) or alternating (black and white) in contrast polarity. In Experiment 2 and 3, report type and contrast polarity were varied in the same way as in Experiment 1, but the task and stimuli differed. Specifically, we measured orientation identification accuracy for stimuli consisting of three randomly rotated Ts (‘tumbling Ts’, Experiment 2), and of three randomly left- or rightward tilted lines (Experiment 3).

The results of Experiment 1 revealed the typical polarity advantage in the single-item report paradigm, with superior performance for the central target when it was of opposite-contrast polarity than the flankers. However, in the full report condition, there was no benefit of alternating relative to uniform contrast polarity. Instead, the effect of stimulus uniformity was inverted, with worse performance in the alternating compared to the uniform condition. The same pattern of results was found in Experiment 2. Hence, the findings of Experiment 1 and 2 revealed an inversion of the target-flanker similarity rule of crowding. When attention was required to multiple instead of a single item, performance with uniform trigrams was superior to alternating trigrams. In Experiment 3, with tilted lines as stimuli, we observed a different pattern of results. Performance in the uniform and alternating condition differed neither when reporting a single line, nor when reporting all lines. Attentional allocation did not modulate performance with simple line stimuli.

Our findings showed that the effect of stimulus uniformity on crowded object recognition was strongly dependent on the attentional selection demanded by the task. Uniformity in the identical contrast polarity conditions was detrimental compared to irregularity in the alternating conditions when a single target was task-relevant, but beneficial when all items required processing. The similarity rule thus no longer held when selective attention was intentionally broadened to include multiple objects. The inversion of one of the central rules of visual crowding by broadened attentional allocation questions the generality of crowding rules. We propose that basic crowding rules do not apply in many real-life situations which, in contrast to typical crowding paradigms, often require broad attention as what constitutes task-relevant targets and irrelevant contexts is usually not pre-defined.

## Experiment 1: letter recognition

### Methods

#### Subjects

Ten students (F = 8, M = 2) between 19 and 27 years of age participated for course credit. All participants reported normal or corrected-to-normal vision. Informed consent was obtained for all participants. Experiments complied with the ethical standards of the Declaration of Helsinki and were approved by the Ethics Committee of the University of Bern.

#### Apparatus

Stimuli were displayed on a 21 inch CRT monitor (HP p1230, refresh rate = 110 Hz, resolution = 1152 × 864). The experiment code ran with Psychopy^59,60^ on a Windows computer. Participants were seated at 57 cm from the screen using a head- and chinrest.

#### Stimuli

**Figure 1:**
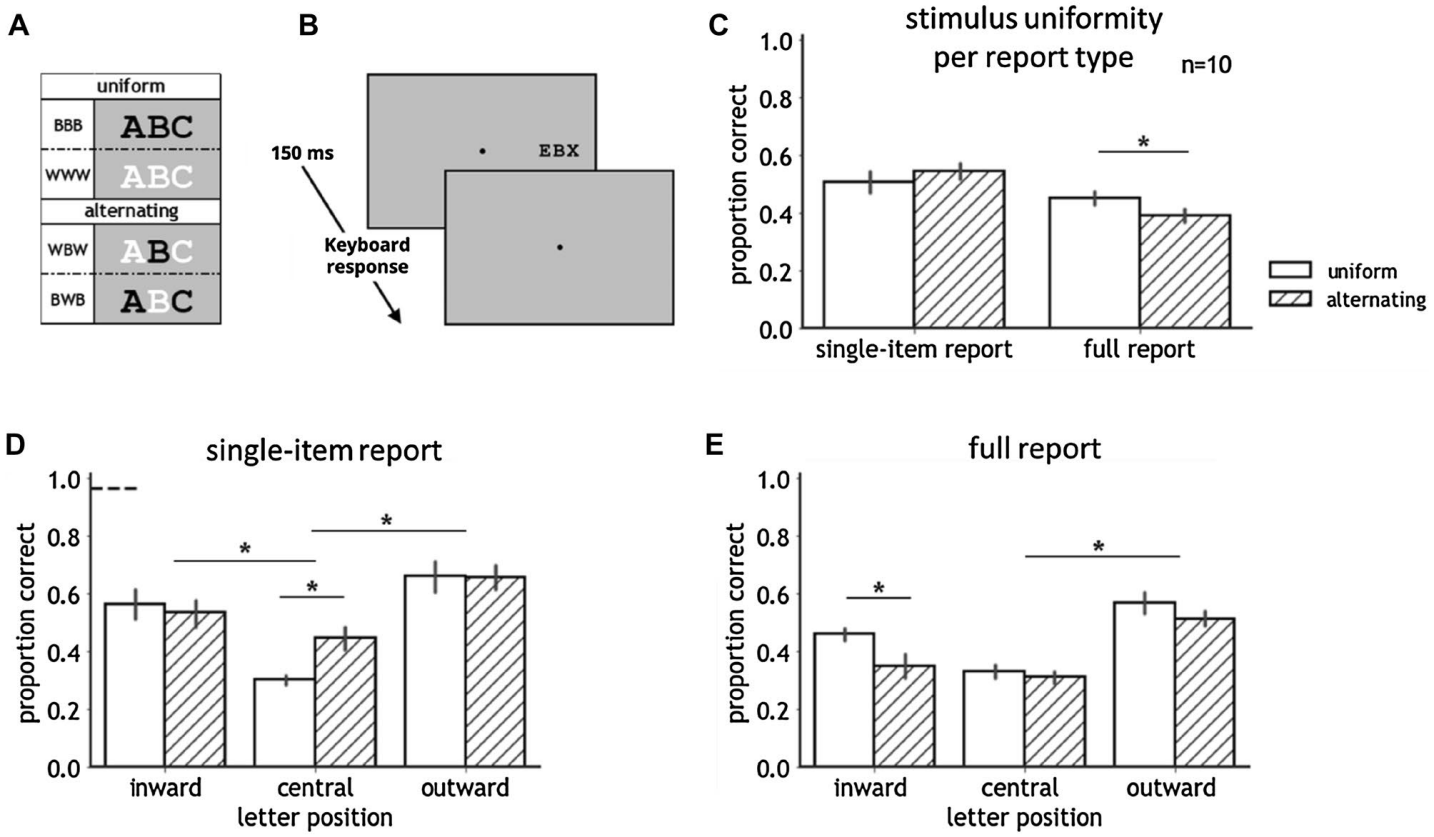
(**A**) Illustration of the letter stimuli in Experiment 1. Trigrams, either uniform or alternating in contrast polarity, consisted of three random, non-repeating letters. In uniform trigrams, all letters had the same contrast polarity (BBB- and WWW-trigrams, with B and W respectively indicating a black and white item), in alternating trigrams the contrast polarity of the central letter was opposite to its adjacent letters (WBW- and BWB-trigrams). (**B**) Time course of a trial for each report type in Experiment 1. Before the first trial of each block, participants were instructed on which letter position(s) to report. (**C–E**) Results of Experiment 1. Asterisks indicate a significant difference at the 0.05 alpha level. Error bars indicate the standard error of the mean. (**C**) Proportion correct for uniform and alternating trigrams in single-item and full report with all letter positions pooled. The interaction between stimulus uniformity and report type was characterized by no difference in proportion correct between uniform and alternating trigrams with single-item report, and worse performance for alternating compared to uniform trigrams with full report. (**D**, **E**) Proportion correct for uniform and alternating trigrams in single-item and full report separately for all letter positions. The dashed line denotes unflanked performance. With single-item report, the polarity advantage was revealed, with better recognition of the central letter in alternating compared to uniform trigrams. With full report, performance for alternating compared to uniform trigrams was worse for the inward letter only.

Stimuli were letter trigrams of three non-repeating letters, randomly drawn from the 26 letters of the alphabet. Trigram letters were all upper case and appeared in the mono-spaced Courier New font. Letters were 1 degree in size, and had a 1.4 degree (center-to-center) spacing between them. Trigrams were centered on the horizontal meridian, with the central trigram letter positioned at ten degrees in the left or right visual field. Trigrams were tested in four contrast polarity conditions (see Fig. 1A). Trigrams consisted of all black letters (0.03 cd/m^2^; BBB-trigrams), all white letters (79.4 cd/m^2^; WWW-trigrams) letters, a black central letter flanked by white letters (WBW-trigrams), or a white central letter flanked by black letters (BWB-trigrams). BBB- and WWW-trigrams were considered uniform, as all letters had the same-contrast polarity; WBW- and BWB-trigrams were considered alternating, as adjacent letters had opposite-contrast polarity. Trigrams were presented on a middle grey (39.6 cd/m^2^) background. For baseline measurement, a single black or white letter was presented at 10 degrees in the left or right hemifield.

#### Procedure

In Experiment 1, we measured recognition accuracy for peripherally presented letter trigrams. Report type and contrast polarity were varied. For each contrast polarity condition, participants reported either a single letter (single-item report) or all letters (full report). In the single-item report condition, at the beginning of each block, observers were instructed whether it was the left, central, or right letter that had to be reported; in full report, participants were instructed to report all three letters from left to right.

Trials were blocked by report type (single-item and full report), letter position (single-item report: inward, central, or outward; full report: inward, central, and outward) and contrast polarity condition (BBB-, WWW-, WBW-, or BWB-trigrams). Overall, there were sixteen different conditions. Observers completed all four contrast polarity conditions of a report type by letter position condition before proceeding to the next block. For example, a participant performed single central letter report for BBB-, WWW-, WBW-, and BWB-trigrams, and then completed all contrast polarity conditions in full report etc. The order of contrast polarity conditions, and report type by letter position conditions was randomized. Each block comprised 20 trials, preceded by four practice trials that were excluded from the analysis. Stimuli were randomly presented in the left or right hemifield, with each block including an equal number of trials (ten) per hemifield. In the first half of the experiment, observers executed every condition once, thus completing 16 blocks or 320 trials. In the second half of the experiment, the condition order was reversed. Additionally, observers performed four blocks of 20 unflanked trials for single black and white letters (two blocks each). Half of the participants performed the unflanked trials in the beginning and end of the experiment, and the other half completed all unflanked trials halfway. Overall, each participant completed 720 trials (640 flanked and 80 unflanked trials). Due to confusions between keyboard layouts QWERTY and QWERTZ (predominantly used in Switzerland), trials with Y or Z both as target and response (i.e., ‘y–z trials’) were excluded for seven participants who showed a proportion of y–z confusion errors above 10 percent on y–z trials (3.4% of all trials).

The procedure of Experiment 1 is shown in Fig. 1B. Each block started with an instruction screen, informing the participant about which trigram letter(s) to report. Next, a black fixation dot appeared, and remained present throughout the trial. Upon key press, a trigram was presented for 150 ms in the left or right visual field. Participants reported the perceived trigram letter(s) by pressing the corresponding keyboard key(s). An auditory feedback signal after each response provided information on registration but not accuracy.

### Results and discussion

To investigate the effect of stimulus uniformity on the recognition of one or multiple crowded letters, the results of the BBB- and WWW-trigrams were combined (i.e., the uniform condition), and the WBW- and BWB-trigrams were combined (i.e., the alternating condition). In single-item report, there was no difference between BBB- and WWW-trigrams (*p* = 0.103), and no difference between WBW- and BWB-trigrams (*p* = 0.855). In full-report, there was no difference between the uniform contrast polarity conditions (*p* = 0.434), and a trend for worse performance for WBW-trigrams compared to BWB-trigrams (*p* = 0.051). Separately for uniform and alternating trigrams, we calculated each participant’s proportion correct for each report type (single-item and full report) and letter position condition (inward, central, and outward). We conducted a repeated-measures ANOVA with accuracy (arcsine-transformed proportion correct) as dependent variable, including report type, stimulus uniformity, and letter position as within-subject variables. All post-hoc comparisons were Tukey-tests. Main effects were revealed for all three factors: letter position (F(2,18) = 15.01, *p* < 0.01, η^2^ = 0.42), stimulus uniformity (F(1,9) = 5.15, *p* < 0.05, η^2^ = 0.002), and report type (F(1,9) = 35.81, *p* < 0.001, η^2^ = 0.11).

Furthermore, our analysis showed an interaction between stimulus uniformity and report type (F(1,9) = 9.47, *p* < 0.02, η^2^ = 0.02). All letter positions taken together, post-hoc Tukey-tests revealed lower performance for alternating compared to uniform trigrams in full report (*p* < 0.02), and no difference in single-item report (*p* = 0.23) (see Fig. 1C). Separately for single-item and full report, we tested for differences between stimulus uniformity conditions at each letter position. In single-item report, we found the typical higher accuracy for the central letter with opposite- compared to same-contrast polarity flankers (*p* < 0.001), and no difference between uniform and alternating trigrams for the other letter positions (inward: *p* = 1.00, outward: *p* = 1.00; see Fig. 1D). In full report, performance for the inward letter was worse for alternating compared to uniform trigrams (*p* < 0.02) (see Fig. 1E). With regard to the remaining letter positions, there was no difference between uniform and alternating trigrams (central: *p* = 1.00; outward: *p* = 0.75). The effect of letter position with single-item report (uniform and alternating trigrams combined) showed worse performance for the central letter compared to both flanking letters (inward: *p* = 0.02; outward: < 0.001). Proportion correct for the inward letter was lower as for the outward letter, but not significantly different (*p* = 0.24). With full report, recognition of the central letter was worse than for the outward letter (*p* < 0.01), and any other differences between letter positions were absent (central vs inward: *p* = 0.57; inward vs outward: *p* = 0.11). Additionally, our results revealed an interaction between letter position and contrast polarity (F(2,18) = 9.59, *p* < 0.01, η^2^ = 0.03). There were no other interactions.

We also analyzed whether the two report types differed in the types of errors observers made. Specifically, we looked at two types of errors: a position error occurred when correctly reporting a stimulus letter but at a false location, and an identity error when reporting a letter not present in the trigram^58^. The proportion of position errors relative to the total number of errors was higher in single-item compared to full report (F(1,9) = 22.62, *p* < 0.001, η^2^ = 0.06). Accordingly, full report had a larger proportion of identity errors compared to single-item report. The prevalence of none of the six possible positions errors differed between report types (all *ps* < 0.22).

As observers were instructed to report all letters from left to right in full report, we also analyzed whether order effects occurred. Letter position was now defined in terms of absolute position (left, central, and right) instead of position relative to fixation (inward, central, and outward). Single-item performance (uniform and alternating trigrams combined) was similar for the left letter compared the right letter (*p* = 0.78), and worse for the central letter compared to both flanking letters (both *ps* < 0.001). In full report, recognition of the left letter was superior compared to the right and central letter (*ps* < 0.001), and did not differ between the right and the central letter (*p* = 0.91).

As expected, in single-item report, recognition of the central letter was worse than of both the inward and outward letter. Superior performance for alternating compared to uniform trigrams when only reporting the central letter confirmed the similarity rule. In full report, none of the letter positions showed a benefit with opposite- relative to same-contrast polarity flankers. Rather, when reporting all letters, performance for alternating trigrams was worse than for uniform trigrams, mainly driven by a significant cost of opposite-contrast polarity for the inward letter position. In sum, the main finding of Experiment 1 showed an inversion of the similarity rule of crowding: alternating compared to uniform contrast polarity flankers facilitated recognition of the central item in single-item report, yet impaired performance for inward and outward letters when reporting all items.

## Experiment 2: orientation identification of tumbling Ts

Experiment 1 revealed the usual superior recognition of a single, central item flanked by opposite- compared to same-contrast polarity flankers, but alternating polarity hindered performance when identifying all trigram letters. In Experiment 2, we investigated whether the same inversion occurred when including stimuli of similar complexity in a 4-AFC orientation task.

### Methods

#### Subjects

Ten participants (F = 7, M = 3) between age 20 and 25 completed the study in return for course credit. They were self-reported non-dyslexics with normal or corrected-to-normal vision, and did not participate in Experiment 1. All participants gave written informed consent.

#### Apparatus and stimuli

**Figure 2:**
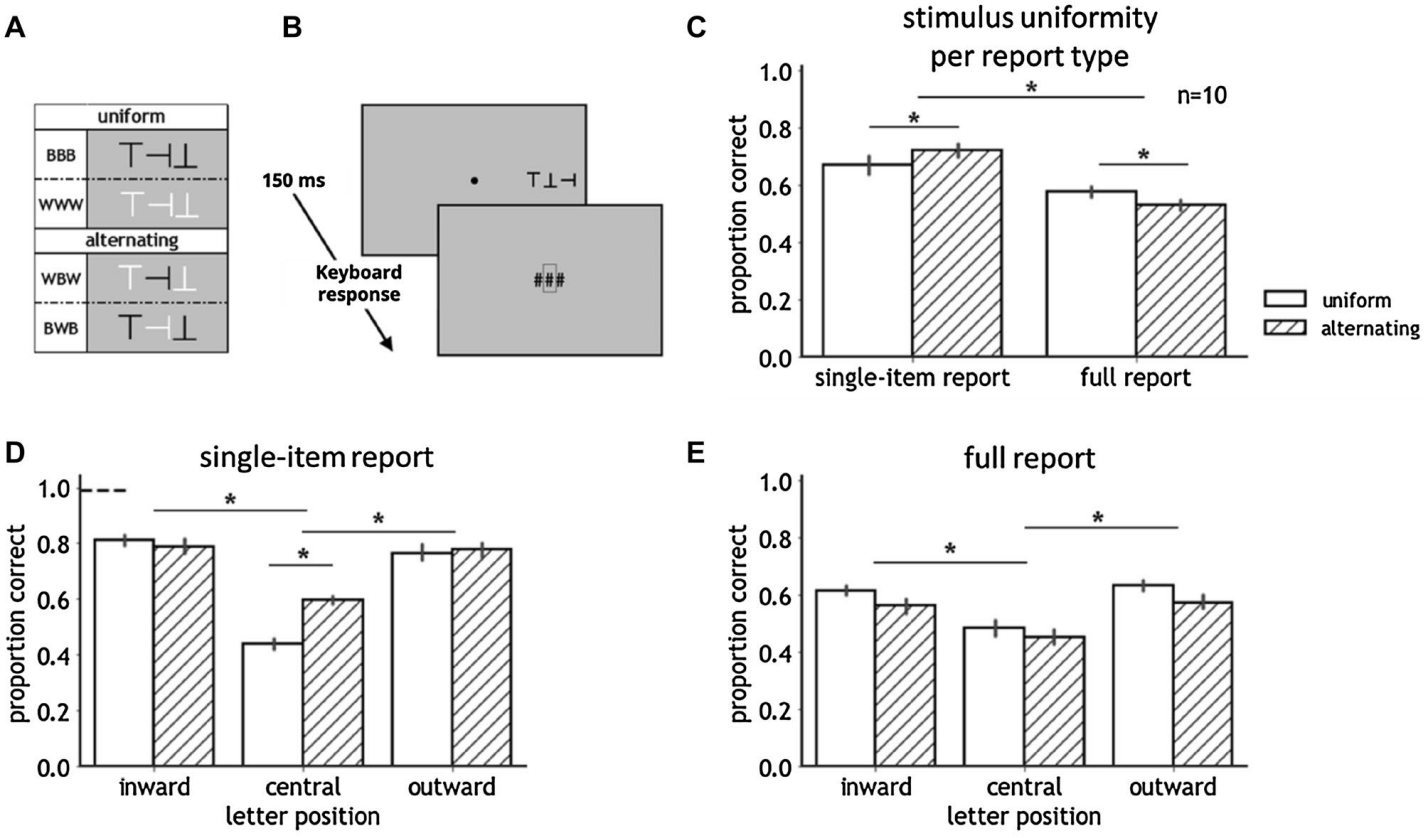
(**A**) Illustration of the stimuli of Experiment 2. Trigrams of ‘tumbling Ts’, either uniform or alternating in contrast polarity, consisted of three Ts of different orientation (0°, 90°,180°, or 270°). BBB, WWW, BWB, and WBW were the four contrast polarity conditions we tested, with B and W respectively representing a black and white item. (**B**) Time course of a trial with single-item report in Experiment 2. The rectangle around the placeholder (‘#’) indicates which letter position required reporting. With full report, the rectangle surrounded all three placeholders. Before the first trial of each block, participants were instructed on which letter position(s) to report. The rectangle and the indicated target position(s) thus remained the same throughout the block. (**C–E**) Results of Experiment 2. Asterisks indicate a significant difference at the 0.05 alpha level. Error bars indicate the standard error of the mean. (**C**) Accuracy for uniform and alternating T-trigrams in single-item and full report with all letter positions pooled. The interaction between stimulus uniformity and report type was characterized by better performance for alternating compared to uniform trigrams in single-item report, and worse performance for alternating compared to uniform trigrams in single-item report. (**D–E**) Accuracy for uniform and alternating T-trigrams in single-item and full report separately for each letter position. The dashed line denotes unflanked performance. With single-item report, the polarity advantage was revealed: Recognition of the central letter was better with opposite- compared to same-contrast polarity flankers. With full report, none of the letter positions showed a significant effect of stimulus uniformity.

Apparatus was identical to Experiment 1. Stimuli consisted of three horizontally aligned rotated Ts, with each T having a unique orientation within the trigram. Rotation of a T was either 0 (upward), 90 (rightward), 180 (downward), or 270 (leftward) degrees, enabling twenty-four possible combinations overall. Each T comprised two orthogonal lines of equal length (1 degree). Adjacent Ts had a center-to-center spacing of 1.4 degrees between them, with the central T presented at 10 degrees in either the left or right visual field. As in Experiment 1, four contrast polarity conditions were included: in the uniform condition, trigrams consisted of all black (BBB-trigram) or all white (WWW-trigram) Ts, and in the alternating condition of a black central T with white flanking Ts (WBW-trigram) or vice versa (BWB-trigram) (see Fig. 2A). Luminance values of stimuli (black: 0.03 cd/m^2^; white: 79.4 cd/m^2^) and background (grey: 39.6 cd/m^2^) were identical to Experiment 1.

#### Procedure

Participants reported the orientation of either a single or all Ts (report type) of a uniform or alternating (stimulus uniformity) T-trigram. As in Experiment 1, report type and contrast polarity were varied. Trials were blocked in an identical manner to Experiment 1, with participants completing 32 blocks of flanked trials and 4 blocks of unflanked trials. The overall number of trials was again 720.

The procedure of Experiment 2 is depicted in Fig. 2B. Similar to Experiment 1, observers were informed on which letter position(s)—either one (left, central, or right) or all Ts (left to right)—required reporting before each block. Stimuli were presented for 150 ms. After stimulus presentation, participants responded with the arrow keys (up, down, left, or right), indicating the orientation of the target T(s). The procedure differed in two ways to Experiment 1. First, after stimulus presentation, the fixation dot was replaced with three placeholders (# # #). A rectangle around either one or all three placeholders repeated which position(s) to report in the current trial. Following the oberver’s response, the relevant placeholder was replaced with a T of the selected orientation. Second, given the visual feedback, no auditory feedback was provided.

### Results and discussion

Data were analyzed identically to Experiment 1. Neither in single-item report (*p* = 0.28) nor in full report (*p* = 0.97), we found different performances for uniform BBB- compared to WWW-trigrams. There was also no difference between WBW- and BWB-trigrams (single-item report: *p* = 1.0; full report: *p* = 0.97). Hence, we combined the results of the BBB- and WWW-trigrams (i.e., the uniform condition), as well as those of WBW- and BWB-trigrams (i.e., the alternating condition). With accuracy (arcsine-transformed proportion correct) as dependent variable, a repeated-measures ANOVA including report type, stimulus uniformity, and letter position as within-subject variables revealed main effects of letter position (F(2,18) = 55.96, *p* < 0.001, η^2^ = 0.42) and report type (F(1,9) = 110.21, *p* < 0.001, η^2^ = 0.26). Performance was worse in full compared to single-item report.

The effect of stimulus uniformity depended on report type (F(1,9) = 23.70, *p* < 0.001, η^2^ = 0.03): accuracy was higher for alternating compared to uniform trigrams in single-item report (*p* = 0.04), yet lower when reporting the orientations of all Ts (*p* = 0.04) (see Fig. 2C). In single-item report, the advantage of alternating over uniform contrast polarity was driven by performance for the central letter (central letter: *p* < 0.001; inward and outward letter: *p* = 1.0) (see Fig. 2D). The lower accuracy for alternating versus uniform contrast polarity in full report was due to absolute differences in the same direction at all letter positions (see Fig. 2E). Overall (uniform and alternating trigrams combined), single-item report was worse for the central letter compared to both flanking letters (inward: *p* < 0.001; outward: *p* < 0.001), and did not differ between inward and outward letters (*p* = 0.82). With full report, our findings showed a similar pattern with worse performance for the central compared to both flankers (inward: *p* < 0.01; outward: *p* < 0.001), and no difference between the inward and outward T (*p* = 0.99). The interaction between report type and letter position (F(2,18) = 13.49, *p* < 0.001, η^2^ = 0.07) was primarily due to better performance in single-item compared to full report for both inward (*p* < 0.001) and outward Ts (*p* < 0.001), with similar accuracies between report types for the central T (*p* = 0.47). Additionally, our findings revealed interactions between contrast polarity and letter position (F(2,18) = 11.29, *p* < 0.001, η^2^ = 0.02), and between all factors of the model (F(2,18) = 7.76, *p* < 0.01, η^2^ = 0.01).

The error analysis showed that the proportion of position errors relative to all errors was higher with single-item compared to full report (F(1,9) = 61.72, *p* < 0.001, η^2^ = 0.07). Full report was thus characterized by a larger proportion of identity errors compared to single-item report. With uniform and alternating trigrams combined, outward letters were perceived more often at the central location in single-item compared to full report (*p* < 0.02), and proportions of the remaining position errors did not differ between report types (all *ps* < 0.36). This pattern differed between uniform and alternating contrast polarity conditions, as indicated by a three way interaction: F(5,45) = 3.34, *p* < 0.02, η^2^ = 0.03).

The analysis of order effects was performed identically to Experiment 1. Single-item performance (uniform and alternating trigrams combined) was similar for the left and right letter (*p* = 0.941), and worse for the central letter compared to both flanking letters (both *ps* < 0.001). In full report, recognition of the left letter was superior compared to the right and central letter (*ps* < 0.001), and did not differ between the right and the central letter (*p* = 0.97).

In single-item report, our data revealed the expected lower performance for the central compared to both flanking Ts. Accuracy did not differ between inward and outward Ts. Similar to Experiment 1, our main finding in Experiment 2 revealed an inversion of the similarity rule of crowding. In single-item report, the similarity rule held: recognition of the central T was superior with adjacent Ts of opposite- compared to same-contrast polarity. However, when all Ts required reporting, worse accuracy for alternating compared to uniform stimuli revealed a violation of the rule of similarity. As in Experiment 1, the findings of Experiment 2 suggested a strong dependence of the effect of target-flanker similarity on report type.

## Experiment 3: orientation identification of tilted lines

Both Experiment 1 and 2 showed an inversion of the similarity rule of crowding. Specifically, alternating contrast polarity improved performance for a single, central target, but deteriorated performance when reporting all stimulus items. In Experiment 1 and 2, stimuli were letters, i.e., complex targets that required integration of multiple features. In Experiment 3, to probe if stimuli of lesser complexity are subject to the same inversion of the target-flanker similarity rule of crowding, we measured orientation identification for tilted lines.

### Subjects

Twelve subjects (F = 8, M = 4) between 20 and 31 years of age participated either for course credit or monetary remuneration. All reported to have normal or corrected-to-normal vision, and gave written informed consent. One subject also participated in Experiment 2.

### Apparatus and stimuli

**Figure 3:**
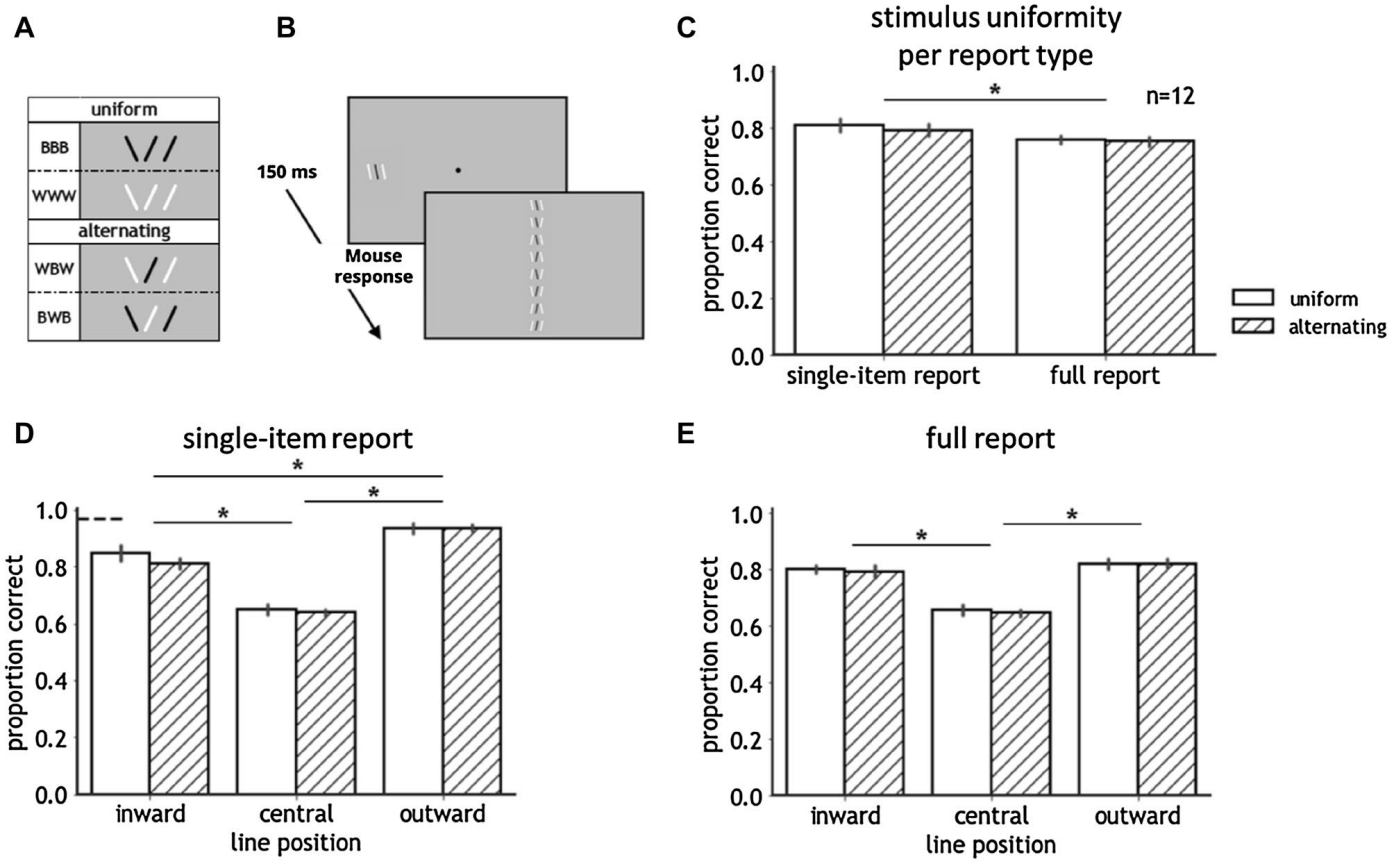
(**A**) Illustration of the stimuli of Experiment 3. Stimuli, either uniform or alternating in contrast polarity, consisted of three lines, each tilted to the left or right (eight possible configurations). BBB, WWW, BWB, and WBW refer to the contrast polarity conditions (‘B’ for ‘black’ and ‘W’ for ‘white’ item). (**B**) Time course of a trial for each report type in Experiment 3. Before the first trial of each block, participants were informed about which line position(s) to report. The lower screen shows the eight response options with full report. With single-item report, only two response options were presented. (**C–E**) Results of Experiment 3. Asterisks indicate a significant difference at the 0.05 alpha level. Error bars indicate the standard error of the mean. (**C**) Accuracy for uniform and alternating lines in single-item and full report with all line positions pooled. (**D**, **E**) Accuracy for uniform and alternating lines in single-item and full report separately for each line position. The dashed line denotes unflanked performance. The polarity advantage was absent, as the single-item report of the central letter was not superior for alternating compared to uniform trigrams. Both with single-item and with full report, none of the letter positions was affected by stimulus uniformity.

Apparatus was identical to Experiment 1 and 2. Stimuli comprised three horizontally arranged, near-vertical lines that were centered on the horizontal midline. Lines were 0.7 degrees long, 0.1 degrees wide, and had 0.35 degrees spacing between them. Each line was randomly tilted 0.1 degrees to the left or right, resulting in eight tilt combinations. Contrast polarity was varied in an identical fashion to Experiments 1 and 2, including uniform BBB- and WWW-triplets and alternating BWB- and WBW-triplets (see Fig. 3A). Luminance values were the same as in the previous experiments (black: 0.03 cd/m^2^; white: 79.4 cd/m^2^; grey background: 39.6 cd/m^2^).

### Procedure

As in Experiment 1 and 2, the independent variables were contrast polarity and report type. We measured accuracy of tilt identification for BBB-, WWW-, WBW-, and BWB-triplets. Participants reported the left- or rightward tilt of one of the three lines (left, central, or right) in single-item report or all three lines in full report. The experimental procedure (see Fig. 3B) was similar to Experiment 1 and 2. At the beginning of each block, an instruction screen informed participants on the line position(s) of which to report the orientation(s). Next, a central fixation dot appeared, and participants initiated the brief stimulus presentation with spacebar. Different from Experiments 1 and 2, the presentation duration was set at 100 ms instead of 150 ms (based on pilot experiments). Furthermore, the response format differed from Experiment 1 and 2. After stimulus presentation, all response options (2-AFC or 8-AFC in single-item and full report, respectively) were displayed one beneath the other, and centered on the screen. Participants responded by selecting the perceived line orientation(s) with a mouse-click. Blocking and counterbalancing was identical to both previous experiments, but the number of trials differed. Participants performed 32 flanked (16 trials each; each tilt combination tested twice) and 4 unflanked blocks (20 trials each), and thus completed 592 trials overall.

### Results and discussion

For both report types, performance differed neither between uniform BBB- and WWW-triplets (single-item report: *p* = 1.0; full report: *p* = 0.97) nor between alternating BWB- and WBW-triplets (single-item report: *p* = 0.96; full report: *p* = 0.87). A repeated-measures ANOVA with accuracy (arcsine transformed proportion correct) as dependent variable, and stimulus uniformity, report type, and line position (inward, central, outward) as within-subject factors revealed main effects of line position (F(2,22) = 140.39, *p* < 0.001, η^2^ = 0.63) and report type (F(1,11) = 24.40, *p* < 0.001, η^2^ = 0.07). Accuracy when reporting all items was worse than in single-item report (see Fig. 3C). Our analysis revealed an interaction between report type and line position (F(2,22) = 0.68, *p* < 0.001, η^2^ = 0.08). With single-item report (uniform and alternating triplets combined), identification of the central line was worse compared to both flanking lines (inward: *p* < 0.001; outward: *p* < 0.001), and performance for the inward line was inferior to the outward line (*p* < 0.001). With full report, accuracy for the central line was lower than for both the inward (*p* < 0.001) and the outward line (*p* < 0.001). Performance did not differ between the inward and outward line (*p* = 0.87). Any other interactions were absent.

Due to the 8-AFC response format in the full report condition, observers were not instructed to report all lines from left to right. Therefore, we did not include an analysis of order effects. Given the presence of repeating line tilts within triplets, neither position nor identification errors were analyzed.

As anticipated, with single-item report, we found worse recognition for the central line compared to both other line positions. Inferior accuracy for inward compared to outward lines indicated inward-outward anisotropy. Surprisingly, we did not find typical uncrowding by opposite-contrast polarity: accuracy for a single central line did not improve with alternating compared to uniform contrast polarity flankers (see Fig. 3D). Contrary to both previous experiments, when identifying all stimulus items, a benefit of uniform compared to alternating contrast polarity was absent (see Fig. 3E). The same experiment, except for a slightly increased presentation duration and line spacing (150 ms and 0.5 degrees respectively) yielded the exact same pattern of results. In brief, the results of Experiment 3 deviated from both previous experiments: performance in single-item report did not replicate the similarity rule of crowding, and there was no inversion of the rule in full report.

## General discussion

The majority of crowding studies uses single-item report paradigms, measuring performance when attentional selection of a single target among task-irrelevant flankers is required. These investigations have revealed, amongst other characteristics, the target-flanker similarity rule of crowding. High target-flanker similarity usually yielded stronger crowding than low target-flanker similarity, which has been shown for a broad range of features, such as color^28,34–36^, depth^28,33,34^, and contrast polarity^28–32^. In typical single-item report paradigms, given the task-irrelevance of the flanking items, performance should benefit (or at least not deteriorate) when not attending the target’s surrounding objects. However, especially with small target-flanker spacings, selective attention towards the target alone may be impaired, and both target and flankers may be compulsory processed together instead^3,61^. In contrast to typical crowding tasks, not attending to contextual items in real-world settings may be less optimal as a priori distinctions between target and context are frequently absent, and surrounding items often carry information about object identity (for reviews see^62–66^). For example, informative contexts have been shown to reduce crowding^67^, as indicated by improved identification of a peripheral object with increasing availability of its typical real-world context. Hence, while optimal performance in crowding tasks usually requires attentional selection of a single item, attentional selection of multiple items may be more appropriate in real-world tasks. Here, we probed the validity of the target-flanker similarity rule when the processing of either a single or multiple crowded items was required. In particular, we investigated whether reduced similarity between neighbouring items would still benefit crowded object recognition in a full report paradigm where broadened attention towards multiple items was required.

Our results revealed an inversion of the similarity rule of crowding. When reporting the central letter only, performance was better for alternating versus uniform letters (Experiment 1 and 2). In full report, opposite-contrast polarity deteriorated crowded letter recognition, with worse performance for alternating compared to uniform polarity letters. In Experiment 3, an orientation discrimination task with tilted lines did not reveal a similar inversion. Instead, performance for uniform and alternating lines was similar at all line positions, both when reporting all line orientations and when reporting the orientation of a single line only. In sum, when decreased target-flanker similarity enhanced performance for a single, central item, reduced similarity between adjacent items was costly when reporting all items.

In Experiment 1 and 2, our findings revealed that report type modulated the effect of target-flanker similarity on performance. Both for letter (Experiment 1) and T-trigrams (Experiment 2), recognition of the central letter was better for alternating compared to uniform trigrams with single-item report, replicating the polarity advantage. However, when reporting all letters, any benefit of alternating over uniform contrast polarity was absent. Instead, our result showed worse performance for alternating compared to uniform trigrams in full report, suggesting benefits of uniformity when all letters were task-relevant. These results are in line with an earlier crowding study that revealed better identification of a central trigram letter when observers had to report all letters instead of the central letter only, suggesting that high-target flanker similarity is less deleterious with full compared to single-item report^57^. Furthermore, with full report, data from a small sample (n = 2) suggested that crowding of the central trigram letter was only minimally stronger when adjacent letters had the same- compared to opposite-contrast polarity flankers^30^. Alternating relative to uniform contrast polarity improved segmentation of a trigram into its constituting letters, but, despite a reduction of crowding, deteriorated performance when the whole stimulus had to be reported. Previous findings have revealed similar uniformity advantages in tasks that also required the identification of multiple letters. For example, we recently showed faster recognition of same polarity words compared to words of which word parts—either syllables or non-syllables—alternated in contrast polarity^32^ (but see ^68^ for beneficial effects of syllable segmentation by color). Deleterious effects of disrupted compared to intact word uniformity were also shown when letters alternated in lower and upper case^69–73^, size^72^ or color^73^. Tasks such as face recognition^74^, vernier offset discrimination^75^, and shape detection^76^ have been shown to benefit from uniformity as well. Hence, in a broad range of tasks, segmenting the initial target object into multiple objects impaired perception of the whole. Here, we showed costs of improved segmentation in a crowding task, questioning the generality of crowding rules.

Experiment 1 and 2 revealed better full report performance when trigrams were uniform, and thus not segmented by alternating contrast polarity. We suggest that the same-contrast polarity letters of uniform trigrams likely formed a more coherent perceptual object than the opposite-contrast polarity letters in alternating trigrams^77^. In foveal vision, experiments on ‘object-based attention’ have shown how attentional deployment is affected by objects (for reviews see^78–80^). In particular, same-object advantages are usually considered to reflect object-based attention. For example, targets were detected faster at an invalidly cued same-object versus different-object location, despite identical distance between the cue and the target locations in both conditions^81^. Previous studies also demonstrated better performance when reporting multiple features of the same object compared to when these features belonged to separate objects (e.g.^82–85^). Same-object advantages have been suggested to reflect a facilitated, often automatic broadening of attention within but not between objects^86–89^ (but see^90^ for strategic control over the spreading of attention within objects), or within uniform versus non-uniform regions^91^. Similar to foveal vision, the difference in ‘objecthood’ between uniform and alternating trigrams may have modulated the attentional spreading of attention in the current study. The superior full-report performance for uniform compared to alternating (non-uniform) trigrams in Experiment 1 and 2 may thus well result from facilitated attentional spreading within uniform but not within alternating trigrams. When selecting a letter in a uniform trigram, automatic spreading of attention to the other same-contrast polarity letters may be beneficial in full report but detrimental in single-item report. Indeed, the automatic spreading of attention within objects has been shown to be deleterious when flankers were irrelevant (e.g.^92–94^). For instance, the categorization of a target letter was worse when incongruent distractor letters had the same color as the target compared to when both had a different color^92^. However, in the alternating trigrams, the attentional processing of all letters is not promoted, which should benefit single-item report but impair full report. Hence, the differential effect of stimulus uniformity between report types in Experiment 1 and 2 can be well explained by effects of unintentional spreading of attention.

The benefit of opposite- compared to same-contrast polarity flankers when reporting only the central item did not result in a similar advantage with full report. Instead, performance was worse for alternating compared to uniform trigrams when reporting all letters. Since low-level properties of uniform and alternating trigrams did not differ between report types, the absence of a polarity advantage in full report cannot be explained based on the stimulus’ features alone, but rather suggests that interference also occurred at a higher level. Although still under debate^44^, higher-level interactions in crowding have been suggested before, for instance, when target letter recognition was impaired more with letter flankers than with number flankers^42,43^. While these studies varied higher-order stimulus properties (e.g., categorical information), we varied task demands in the current study. Modulations of crowding by task demands were revealed earlier, with worse identification of a crowded target defined by form than by category^95^ or different effects of target-flanker spacing in an identification compared to a magnitude comparison task^96^. In the current study, depending on the task demands, observers had to attend one or three items. As the size of the attentional window has been shown to be optimized in function of the task goal^97^, the spatial deployment of attention likely varied between report types in the current study, with more focused attention in single-item report and more diffused attention in full report. Importantly, differences in the size of the attentional window have been shown to override key properties of crowding. Previously, inward-outward anisotropy was revealed under focused but not diffused attention^19^: An outward flanker was more deleterious than an inward flanker when the target appeared at the same eccentricity on each trial, but inward and outward flankers were equally deleterious when the target could appear at one of three possible eccentricities. Here, the locus of attention seems to have modulated another signature characteristic of crowding, namely the similarity rule. While our current results show strong attentional modulations of crowding, our main findings do not allow for strong claims regarding the overall role of attention in crowding.

In Experiment 3, identification of the single, central line was not better when flanking lines had opposite- compared to same-contrast polarity. Instead of the usual polarity advantage, our results revealed similar performance levels for uniform and alternating triplets when identification of only the single, central line was needed. Since opposite- compared to same-contrast polarity reduced crowding with more complex letters (Experiment 1 and 2) but not with simple lines (Experiment 3), this might suggest a role of stimulus complexity. However, the polarity advantage has been previously shown with stimuli of limited complexity, similar to our line stimuli. For example, discriminating the offset for a (vertical) vernier was superior with vertical flanking lines of opposite- compared to same-contrast polarity^34^. Instead of stimulus complexity, we propose that configural grouping between target and flanking lines might account for the absence of the polarity advantage when reporting the central line only. Recently, we showed better tilt discrimination for the central line within alternating compared to uniform triplets with bidirectional flankers (\/ or /\) but similar performance with unidirectional flankers (\\ or //)^53^. When examining the dependence of the polarity advantage on flanker tilt in a follow-up experiment, our findings revealed a larger configural superiority effect for uniform versus alternating triplets with unidirectional flankers, suggesting that emergent features benefitted performance for uniform triplets more than for alternating triplets. Taken together, the absence of the polarity advantage for triplets with unidirectional flankers in that study indicated that emergent features between same-contrast polarity lines benefitted performance to the extent that the performance level was similar as with opposite-contrast polarity flankers. In the current study, emergent features may explain the absence of the polarity advantage when reporting the central line tilt only as well. In particular, the configurations formed by two adjacent lines, either parallel (\\ or //) or mirrored (/\ or \/), may have provided task-relevant information to perform the line orientation task. Similar emergent features were associated with improved performance in a crowding task, and were shown to override conventional crowding rules^23,52^. For instance, when a diamond-like shape was better recognized when flanked by diamonds compared to Xs, a key role in this exception to the similarity rule was attributed to the emergent feature of closure^23^. In full report, accuracy for uniform lines possibly benefitted from strong task-relevant grouping, yet suffered from more severe crowding with same-contrast polarity lines. For alternating lines, reduced crowding by alternating contrast polarity was supposedly counteracted by weaker task-relevant grouping and costs of disrupting uniformity (e.g.^32^). Hence, both with single-item and full report, the interplay between crowding and configural grouping may explain the similar performance for uniform and alternating lines. Importantly, our findings suggest—instead of the usual cost—an advantage of strong target-flanker grouping when providing a surplus in task-relevant information.

Our key results (Experiment 1 and 2) showed that stimulus uniformity was detrimental when attentional selection of only a single element was required, but beneficial when attention was broadened to all stimulus’ elements. These findings suggested that the effect of similarity-based perceptual grouping is strongly dependent on how observers have to direct their attention in order to meet task demands. With stimuli of lesser complexity, Experiment 3 revealed neither the similarity rule in single-item report nor the inversion in full report. We attributed a crucial role in the divergent outcome with line triplets to the task-relevant information provided by configural grouping between adjacent lines. Taken together, our results provide further evidence for a strong association between attention and crowding. The reversal of the similarity rule found under broad attention puts strong constraints on the validity of basic crowding rules in real-life contexts. A well-established crowding rule, which was predominantly revealed when the recognition of a single object amongst task-irrelevant flankers was needed, was no longer valid when the task required the attentional selection of multiple items. We propose that even key properties of crowding are not rigid but are instead strongly dependent on the attentional demands imposed by the task.
